# The Fidelity of Training in Behaviour Change Techniques to Intervention Design in a National Diabetes Prevention Programme

**DOI:** 10.1007/s12529-021-09961-5

**Published:** 2021-02-09

**Authors:** Rhiannon E. Hawkes, Elaine Cameron, Lisa M. Miles, David P. French

**Affiliations:** 1 Division of Psychology and Mental Health, Manchester Centre of Health Psychology, University of Manchester, Coupland 1 Building, Manchester, M13 9PL UK; 2 Division of Psychology, University of Stirling, Stirling, Scotland, UK

**Keywords:** Behaviour change techniques, Fidelity, Staff training, Diabetes prevention, Type 2 diabetes

## Abstract

**Background:**

The National Health Service Diabetes Prevention Programme (NHS-DPP) is a behavioural intervention for people identified as high risk for developing type 2 diabetes that has been rolled out across England. The present study evaluates whether the four commercial providers of the NHS-DPP train staff to deliver behaviour change technique (BCT) content with fidelity to intervention plans.

**Method:**

One set of mandatory training courses across the four NHS-DPP providers (seven courses across 13 days) was audio-recorded, and all additional training materials used were collected. Recordings and training materials were coded for BCT content using the BCT Taxonomy v1. BCTs and depth of training (e.g. instruction, demonstration, practice) of BCT content was checked against providers’ intervention plans.

**Results:**

Ten trainers and 78 trainees were observed, and 12 documents examined. The number of unique BCTs in audio recordings and associated training materials ranged from 19 to 44 across providers, and staff were trained in 53 unique BCTs across the whole NHS-DPP. Staff were trained in 66% of BCTs that were in intervention plans, though two providers trained staff in approximately half of BCTs to be delivered. The most common way that staff were trained in BCT delivery was through instruction. Training delivery style (e.g. experiential versus educational) varied between providers.

**Conclusion:**

Observed training evidences dilution from providers’ intervention plans. NHS-DPP providers should review their training to ensure staff are trained in all key intervention components, ensuring thorough training of BCTs (e.g. demonstrating and practicing how to deliver) to enhance BCT delivery.

## Introduction

In 2004, the World Health Organization reported that worldwide incidence of type 2 diabetes had increased to 422 million people [1]. In the United Kingdom (UK), 3.8 million adults were reported to have the condition in 2015, with a projected increase to 4.9 million by 2025 [2]. In response to this, the National Health Service Diabetes Prevention Programme (NHS-DPP) was launched by Public Health England in 2016. This is a behavioural intervention for adults with elevated blood glucose levels, or non-diabetic hyperglycaemia, to slow or stop their progression to type 2 diabetes [3]. The NHS-DPP is one of several diabetes prevention programmes internationally, which have prevented progression from non-diabetic hyperglycaemia to type 2 diabetes in attendees [4, 5]. Early results suggest that the NHS-DPP has been effective in achieving these outcomes [6]. During the first three waves of implementation of the programme in 2016–2019, the NHS-DPP was delivered by four independent provider organisations who each secured contracts to deliver the service in localities across England [7].

The main aims of the programme were to bring about weight loss and reduced blood glucose levels through delivery of behaviour change techniques (BCTs) to change dietary and physical activity behaviours [8]. BCTs are the ‘active ingredients’ of interventions that produce behavioural change in individuals [9]. Nineteen core BCTs were stipulated for inclusion in diabetes prevention programmes by an evidence review underpinning the NHS-DPP intervention [25]. The majority of these BCTs were designed to improve self-regulation of behaviours [10], through prompting goal setting, action planning, problem solving and self-monitoring. NHS England specified that staff employed by providers should be sufficiently trained in the delivery of the service and behaviour change content [8].

A fidelity assessment of the NHS-DPP is critical to establish reasons for its effectiveness, or lack thereof, and whether its benefits are comparable to the published trials in reducing the onset of type 2 diabetes. Intervention fidelity describes whether an intervention was delivered as intended [11]. Without adequate assessment of fidelity, it cannot be ascertained whether intervention effectiveness is due to the intervention being implemented as planned, or if it is due to other factors added to or omitted from the intervention [12]. The National Institutes of Health Behaviour Change Consortium (NIH-BCC) model defines five domains of assessing fidelity at each stage of an intervention. These are study design (whether the planned intervention is in line with underlying theory), provider training (whether deliverers are trained in key components of the planned intervention), treatment delivery (whether the intervention’s key components are actually delivered), treatment receipt (whether recipients understand the intervention) and treatment enactment (whether recipients incorporate the key components of the intervention in their day-to-day lives) [11].

Previous fidelity assessments have mainly focused on fidelity of delivery. For example, in trials of complex healthcare interventions, treatment delivery is the most common component of fidelity measured, as reported by 96% of surveyed researchers [13]. Further, in routine practice as opposed to research studies, fidelity evaluations of health interventions are less common, but still have a focus on fidelity of delivery (e.g. the NHS England Stop Smoking Service [14]). However, if the other domains of the NIH-BCC model are not accounted for, evaluators should have less confidence in drawing accurate conclusions about what happened in an intervention and why. For example, if staff training for a programme is poor, it is likely that the delivery of the programme will also be poor, which should subsequently lead to poor receipt of the intervention [15]. Without a fidelity assessment of training, evaluators will be less certain about the solutions to enhance fidelity of delivery. For that reason, researchers recommend encompassing the whole fidelity model, and where feasible, considering each of these five domains of fidelity [15].

To date, a handful of studies have assessed training fidelity in various settings, including dementia care [16, 17], ‘train the trainer’ model in suicide prevention [18] and a physical therapy intervention [19]. In relation to health-related behaviours specifically, a systematic review evaluated fidelity assessments in physical activity interventions and found only two studies reported assessment of training fidelity, but noted a lack of clear distinction between fidelity of training and fidelity of delivery [20]. Other work has looked at how to optimise staff training using fidelity strategies in interventions to increase physical activity in those with type 2 diabetes [21, 22], though this work did not include an assessment of training fidelity specifically.

Adequate training of intervention deliverers provides them with information about the theory underpinning the programme and the necessary skills required for the intervention, therefore ensuring competence across deliverers [23]. However, this includes not only providing intervention deliverers with adequate knowledge, but also showing them how to deliver key intervention features (‘showing how’) and allowing them to demonstrate these new skills by ‘doing’ [24]. Thus, when staff are being trained in delivering behaviour change content, as is the case in the NHS-DPP, this model [24] suggests that they should not only be told which BCTs to deliver, but also shown how to deliver BCTs for specific activities and given the opportunity to practice BCT delivery.

In line with the NIH-BCC guidance [11], the authors of the current paper have previously evaluated fidelity of providers’ planned BCTs to BCTs specified in the evidence base [8, 25]. This evaluation of the NHS-DPP study design found that providers planned to deliver 74% of the BCTs that an evidence review indicated [26]. There are no studies assessing fidelity of staff training in diabetes prevention programmes internationally, despite trials being implemented in multiple countries [4, 5, 27–29]. For the NHS-DPP in England, a formal fidelity assessment was not conducted in the previous evaluation of the pilot in 2015 or subsequent phased roll-out in 2016 [30]. Further, to the authors’ knowledge, there are no other formal fidelity assessments of staff training in multi-site public health interventions, and there are none that focus on training behaviour change techniques. A fidelity assessment of staff training of BCTs in the NHS-DPP is important because if lack of fidelity in the delivery of the programme is detected, it needs to be clear whether this is due to ineffective training or other contextual factors in the delivery of the intervention.

The aims of the current study were to (1) describe the behaviour change content of staff training across the four provider organisations delivering the NHS-DPP, (2) evaluate fidelity of BCT training to the four providers’ intervention plans, and (3) describe the depth of BCT training and the delivery style of staff training. This paper provides a unique and comprehensive evaluation of the fidelity of BCTs that staff were trained to deliver in the NHS-DPP compared to providers’ intervention plans.

## Methods

### Design and Participants

An observational study comparing the mandatory staff training from each of the four NHS-DPP providers to those providers’ intervention plans. The four NHS-DPP providers were commercial organisations who each secured contracts to deliver the NHS-DPP in localities across England in 2018–2019.

Trainers were employed by each of the four providers or the intervention developers and delivered the training to newly appointed facilitators employed by each of the providers via face-to-face group training courses. Trainee facilitators were required to attend mandatory training courses on the group delivery of the NHS-DPP as part of their induction to the job role before they were allocated their own groups to deliver in the field. The appointed trainee facilitators were not health professionals, but they came from backgrounds including nutrition, personal training and public health.

### Procedures

Researchers attended one set of mandatory staff training for each of the four providers between February and December 2018. The training sampled was based on the timing of training of each NHS-DPP provider who were recruiting new staff and delivering staff training at the time of the evaluation (2018–2019). The four provider training courses were observed in four different geographical areas. Written informed consent was obtained from participants prior to the training session starting and prior to researchers turning on the audio-recorder. Participants consented to researchers taking notes on the content of the training sessions.

An audio-recorder was placed next to the trainer at the front of the room to capture all training content, including BCTs, delivered during the training sessions, and a new file was used for each 30–120-min session throughout the day.

Researchers requested the pre-course reading materials supplied to trainees from the management staff employed by each of the providers with whom researchers were in contact with. Such documentation was either sent via e-mail or hard copies were posted to the research team. The research received ethical approval by the North West – Greater Manchester East NHS Research Ethics Committee on 1 August 2017 (Reference: 17/NW/0426).

### Materials

Documents detailing providers’ intervention plans were obtained prior to researchers observing the NHS-DPP staff training sessions. These consisted of the following for each provider:

- Framework response bids describing the proposed service delivery which were submitted by providers during service procurement
- Programme manuals containing a session-by-session protocol for facilitators to follow when delivering the programme.

Assessment of training content consisted of the following for each provider:

- Audio-recordings of NHS-DPP staff training courses (*n* = 47 audio recordings captured across 13 training days observed across all four providers)
- Additional researcher field notes written during each training session, capturing any other notable observations such as other training content covered (e.g. training of group facilitation behaviours, group management) and delivery style of training (e.g. the use of educational materials, role play, the general rapport and interaction between trainers and trainees, and the types of discussions covered during the training)
- All pre-course reading materials that were distributed to trainees prior to each of the training courses, e.g. pre-training handbooks, journal articles

Initial assessments (a consultation which service users attended prior to being enrolled onto the NHS-DPP group sessions) were not part of the formal behaviour change intervention as they determined eligibility for the group sessions [8], and not all providers trained staff in how to deliver initial assessments (as sometimes it was sub-contracted out to another healthcare professional). Therefore, we did not include the initial assessment protocols within the main fidelity analysis, although sensitivity analyses were conducted and detailed in the results.

### Analyses

BCT coding used the Behaviour Change Technique Taxonomy v1 (BCTTv1; [9]), defining 93 distinct BCTs. Coding was documented using author-developed data collection forms (see Electronic Supplementary Material 1 for the data collection checklist used in the staff training observations and Electronic Supplementary Material 2 for BCT coding instructions). Researchers underwent training in the use of the BCTTv1 [32] and a set of coding rules were developed through team discussions following guidance from taxonomy authors. Coding rules were based on those previously used to code providers’ intervention plans [26].

Intervention plans comprised each provider’s programme manuals and framework response bid combined, as these documents gave the most comprehensive description of the BCTs that providers planned to include in their programmes. BCTs identified in the NHS-DPP intervention plans are reported elsewhere [26]. These ‘design’ criteria were compared with the staff training of BCTs identified in the audio-recordings and associated training materials. Assessment of the BCTs in the NHS-DPP intervention plans demonstrated moderate to strong agreement between coders [31] (kappa values ranged from 0.75 to 0.88; [26]).

Researcher REH independently coded all training materials that were supplied by each provider and the audio-recordings of each providers’ staff training courses for BCTs that staff were trained to deliver. A new instance of a BCT was coded when a new intervention activity was described or if a different health behaviour (e.g. diet, physical activity) was targeted. The level of target behaviour was also documented when coding the BCT ‘information about health consequences’ (e.g. levels of the target behaviour ‘diet’ included information about carbohydrates, fats, sugar, etc.) as researchers felt these were distinct pieces of information targeting distinct behaviours.

Researcher LMM double-coded 10% of the audio-recordings of staff training sessions (*n* = 5 audio recordings from training courses). Interrater reliability (IRR) was calculated using the kappa statistic to determine consistency between coders [31]. Identified coding discrepancies were discussed between REH, LMM and DPF until agreement was met.

The depth of BCT training was also coded to capture the varying methods in which staff were trained in specific BCTs. One of the following labels was given to each of the BCTs coded in the face-to-face staff training sessions:

- Informed about BCT (e.g. background reading and providing background information about the BCT)
- Directed to deliver BCT (e.g. the BCT is mentioned or referred to when describing an intervention activity)
- Instructed how to deliver BCT (trainer delivers instruction on how the BCT can be delivered in group sessions)
- Demonstrated how to deliver BCT (trainer demonstrates the delivery of the BCT; e.g. demonstrates how to conduct a problem solving activity)
- Practiced how to deliver BCT (trainees practice delivering BCT, e.g. trainees practice delivering a problem solving activity)
- Modelled how to deliver BCT (the training delivery models the intervention delivery so trainees could experience the BCT from the patients’ perspective, e.g. role play of a problem solving activity so trainees can experience participating in this activity)

To assess extent of staff training fidelity to intervention plans, the BCTs in training courses and pre-course reading were compared to the BCTs present in each of the four providers’ intervention plans (programme manuals and framework response documents). The proportion of additional BCTs that staff were trained in but were not specified in provider’s intervention plans was also calculated.

## Results

### Study Sample

Each provider had a different number of mandatory training courses that staff were required to attend, which lasted between 2 and 5 days depending on the provider delivering the training. The final sample of NHS-DPP staff training consisted of seven mandatory training courses (two training courses for provider A, three training courses for provider B, one each for providers C and D). All attending trainers (*n* = 10) and trainees (*n* = 78) consented to the researchers attending, observing and audio-recording the NHS-DPP staff training courses. Both trainers and trainees had a diversity of backgrounds (see Table 1).

**Table 1:** Number of mandatory training courses and participants consented for each provider

	Mandatory training courses	Total length of training	Participants recruited (*n*)	Trainer backgrounds	Trainee backgrounds
Provider A	2 training courses	3 days	3 trainers 15 trainees	Nutrition, dietetics, private healthcare	Health promotion, cardiac rehabilitation, nutrition, personal trainer
Provider B	3 training courses	5 days	5 trainers 51 trainees	Personal trainer, public health, nutrition	Health psychology, personal trainer, nutrition, mental health
Provider C	1 training course	3 days	1 trainer 9 trainees	Public health	Sports science, personal trainer, wellbeing practitioner, nutrition
Provider D	1 training course	2 days	1 trainer 3 trainees	Diabetes specialist	Health psychology, nutrition, nutritionist
	Overall no. of training courses: 7 Overall no. of training days: 13		Overall total no. of participants: 88		

### BCTs Present in Staff Training

Staff were trained in a total of 53 unique BCTs across the four providers. Each provider trained staff on between 19 and 44 unique BCTs in their face-to-face training and accompanying pre-course reading. Kappa values ranged from 0.61 to 0.80 for the staff training sessions, demonstrating moderate to strong agreement between coders [31], prior to resolving discrepancies (see Table S1 in Electronic Supplementary Material 3 displaying IRR values for providers’ intervention plans and Table S2 in Electronic Supplementary Material 3 displaying IRR values for the staff training sessions).

In the face-to-face training courses, the number of distinct instances of training in the use of specified BCTs that occurred were 72, 207, 292 and 57 times, respectively, for providers A, B, C and D. For three of the providers, staff were most commonly trained in the BCT ‘Information about health consequences’, trained 23 times with provider A, 73 times with provider B and 45 times with provider C. For provider D, staff were most commonly trained in the BCT ‘Social support (unspecified)’, trained 11 times, followed by ‘Information about health consequences’, trained 10 times.

Overall, there were 11 BCTs in which all staff were trained across providers: action planning, behavioural practice, behavioural substitution, goal setting for behaviours, goal setting for outcomes, information about health consequences, information about emotional consequences, problem solving, self-monitoring of behaviours, self-monitoring of outcomes and unspecified social support (see Electronic Supplementary Material 4 for BCT definitions, according to the BCTTv1; [9]). Ten of these BCTs had been recommended for inclusion in intervention delivery in the NHS commissioning specification [8] or public health guidelines [25].

### Fidelity of Trained BCTs to Intervention Design

The BCTs present in the staff training of each provider (pre-course reading and training courses) were compared to the planned BCTs in each providers’ intervention plans (framework responses and programme manuals; see Table 2). Sixty unique BCTs were specified in the intervention plan documents across all four providers. Providers B and C had the highest fidelity of BCTs delivered in staff training (81.6% and 85.1%, respectively), whereas providers A and D only trained staff in approximately half of BCTs they were planning to deliver (46.3% and 51.3%, respectively). Overall, fidelity of the intervention training to the planned intervention was 66.1%.

**Table 2:** Unique BCTs specified in providers’ intervention plans compared to BCTs present in each providers’ staff training

Behaviour change techniques	A design	A training	B design	B training	C design	C training	D design	D training
*Action planning*	✓	✓	✓	✓	✓	✓	✓	✓
*Behaviour substitution*	✓	✓	✓	✓	✓	✓	✓	✓
*Behavioural practice/rehearsal*	✓	✓	✓	✓	✓	✓	✓	✓
*Credible source*	✕	✕	✕	✓	✕	✕	✓	✕
*Feedback on behaviour*	✓	✓	✓	✓	✓	✓	✓	✕
*Goal setting (behaviour)*	✓	✓	✓	✓	✓	✓	✓	✓
*Goal setting (outcome)*	✓	✓	✓	✓	✓	✓	✓	✓
*Graded tasks*	✓	✕	✕	✓	✓	✓	✓	✓
*Information about health consequences*	✓	✓	✓	✓	✓	✓	✓	✓
*Monitoring of outcome(s) of behaviour without feedback*	✓	✕	✓	✕	✓	✓	✓	✕
*Pharmacological support*	✓	✕	✓	✕	✕	✕	✕	✕
*Problem solving*	✓	✓	✓	✓	✓	✓	✓	✓
*Pros and cons*	✓	✕	✕	✓	✓	✓	✓	✕
*Review outcome goal(s)*	✓	✕	✓	✓	✓	✓	✓	✓
*Self-monitoring of behaviour*	✓	✓	✓	✓	✓	✓	✓	✓
*Self-monitoring of outcome(s) of behaviour*	✓	✓	✓	✓	✓	✓	✓	✓
*Social support (emotional)*	✓	✕	✓	✓	✕	✓	✓	✓
*Social support (practical)*	✓	✕	✓	✕	✓	✓	✓	✓
*Social support (unspecified)*	✓	✓	✓	✓	✓	✓	✓	✓
Adding objects to the environment	✓	✓	✓	✓	✓	✓	✓	✓
Avoidance/reducing exposure to cues for the behaviour	✓	✓	✓	✓	✓	✓	✓	✕
Biofeedback	✓	✕	✓	✓	✓	✓	✓	✓
Commitment	✓	✕	✓	✓	✓	✓	✕	✕
Comparative imagining future outcome	✓	✕	✕	✕	✕	✕	✓	✓
Demonstration of behaviour	✓	✓	✓	✓	✓	✕	✓	✕
Discrepancy between current behaviour and goal	✓	✕	✕	✕	✕	✕	✓	✕
Distraction	✕	✕	✕	✕	✓	✓	✕	✕
Feedback on outcome(s) of behaviour	✓	✕	✓	✓	✓	✓	✓	✓
Focus on past success	✓	✕	✓	✓	✓	✕	✓	✕
Framing/reframing	✕	✕	✓	✓	✓	✓	✓	✕
Habit formation	✓	✕	✓	✕	✓	✕	✕	✓
Identification of self as role model	✕	✕	✕	✕	✕	✕	✓	✕
Increase positive emotion^a^	✕	✕	✓	✓	✕	✕	✓	✓
Incentive (outcome)	✕	✕	✕	✕	✓	✓	✕	✕
Information about antecedents	✓	✓	✓	✓	✓	✓	✓	✕
Information about emotional consequences	✓	✓	✓	✓	✓	✓	✓	✓
Information about social and environmental consequences	✓	✕	✓	✕	✓	✓	✕	✕
Instruction on how to perform the behaviour	✓	✓	✓	✓	✓	✕	✓	✕
Material incentive (behaviour)	✕	✕	✕	✕	✕	✓	✕	✕
Material reward (behaviour)	✕	✕	✕	✕		✕	✕	✕
Mental rehearsal of successful performance	✕	✕	✓	✓	✓	✓	✓	✕
Non-specific reward	✕	✕	✕	✕	✕	✓	✕	✕
Overcorrection	✕	✕	✕	✕	✕	✕	✓	✕
Prompts/cues	✓	✓	✓	✕	✓	✓	✕	✕
Reduce negative emotions	✓	✕	✓	✓	✓	✓	✓	✕
Remove access to the reward	✕	✕	✕	✕	✓	✓	✕	✕
Remove reward	✕	✕	✕	✓	✕	✕	✕	✕
Restructuring the physical environment	✕	✕	✓	✓	✓	✓	✕	✕
Restructure the social environment	✕	✕	✕	✕	✓	✓	✕	✕
Review behaviour goal(s)	✓	✕	✓	✓	✕	✓	✓	✕
Reward (outcome)	✓	✕	✕	✕	✓	✓	✕	✕
Salience of behaviours^b^	✓	✓	✕	✕	✓	✓	✓	✓
Salience of consequences	✓	✕	✓	✓	✓	✕	✕	✓
Self-incentive	✕	✕	✓	✕	✓	✕	✕	✕
Self-reward	✓	✕	✓	✓	✓	✓	✕	✕
Self-talk	✕	✕	✕	✕	✓	✓	✓	✕
Social comparison	✓	✕	✕	✕	✓	✓	✕	✕
Social incentive	✕	✕	✕	✕	✓	✓	✕	✕
Social reward	✓	✕	✓	✓	✓	✓	✓	✕
Verbal persuasion	✕	✕	✕	✕	✕	✕	✓	✕
BCTs planned in intervention design	41		38		47		39	
Planned BCTs included in staff training	19		31		40		20	
Fidelity of staff training to intervention design (%)	46.3		81.6		85.1		51.3	
Planned BCTs not included in staff training	22		7		7		19	
Unplanned BCTs included in staff training	0		5		4		2	

BCTs present in providers’ intervention plans include those in the framework response documents and programme manuals combined for each provider. BCTs in staff training include those present in face-to-face training and pre-course reading materials. BCTs in italics are those 19 core BCTs specified in the evidence base underpinning the NHS-DPP [26]

^a^Increase positive emotions is not listed in the BCTTv1, but was noted by the authors for inclusion in the next version of the taxonomy

^b^Salience of behaviours was not listed in the BCTTv1, but has been identified as a new behaviour change technique by the authors of this paper

The number of BCTs omitted in staff training varied between providers; 22, 7, 7 and 19 BCTs were omitted in the staff training that were present in the intervention plans of providers A–D, respectively. There were also some additional BCTs delivered in the training which were not present in providers’ intervention plans, ranging from no additional BCTs in provider A’s training to five additional BCTs delivered in provider B’s training (see Table 2). Electronic Supplementary Material 5 shows some sensitivity analyses, comparing trained BCTs to providers’ individual framework response documents and programme manuals separately. There were minimal differences in fidelity scores between framework response and programme manual for providers B and C. Providers A and D had higher fidelity to their programme manuals.

### Depth of BCT Training

For each provider, training for the majority of BCTs was face-to-face, although provider D trained 23% of BCTs in materials alone. Across all four providers, the most common way that staff were trained in BCTs during the face-to-face courses was by instructing them how to deliver a BCT (see Table 3). However, there were some BCTs which staff were only either ‘informed about’ or ‘directed to deliver’, but with no further elaboration on how these BCTs should be delivered in a group session. Staff were only ‘informed about’ or ‘directed to deliver’ a total of three (15.8%), five (14.7%), six (14.6%) and two (11.8%) unique BCTs with providers A–D, respectively. This included some self-regulatory BCTs, for example, provider A only directed staff to deliver ‘action planning’ and providers B and D only informed or directed trainees about ‘self-monitoring of behaviour’, with no further training on how to deliver these BCTs. Tables S7–S10 in Electronic Supplementary Material 6 provides a further breakdown of which behaviours were targeted for each BCT trained by each of the providers, and the depth of training for each BCT targeting each health behaviour.

**Table 3:** Number of BCTs trained and depth of BCT training across each providers’ face-to-face training courses

	Provider A		Provider B		Provider C		Provider D	
No. of BCTs in training (face-to-face and materials)	19		36		44		22	
	*n*	%	*n*	%	*n*	%	*n*	%
Unique BCTs trained face-to-face	19	90.5	34	94.4	41	93.2	17	77.3
Depth of BCT delivery^a^								
Unique BCTs informed about	3	15.8	3	8.6	7	16.7	0	0.0
Unique BCTs directed to deliver	5	26.3	20	60.0	22	53.7	8	47.1
Unique BCTs instructed how to deliver	11	57.9	24	68.6	30	71.4	10	58.8
Unique BCTs demonstrated how to deliver	1	5.3	14	40.0	10	23.8	6	35.3
Unique BCTs practiced how to deliver	10	52.6	1	2.9	2	4.8	6	35.3
Unique BCTs modelled	10	52.6	6	17.1	9	21.4	6	35.3

^a^Some unique BCTs were delivered more than once via different modes of delivery (e.g., trainers may have directed to deliver a unique BCT, and later demonstrated how to deliver that same BCT). This has been captured in the above table

 Despite providers A and D training staff in fewer BCTs overall in their face-to-face training (19 and 17 unique BCTs, respectively), a higher proportion of trained unique BCTs were practiced by trainees and modelled actual delivery (i.e. trainers delivered training using a desired BCT so trainees could experience the BCT from the patients’ perspective. For example, trainer might do a problem solving activity around how to manage difficult conversations so trainees can experience participating in a problem solving activity), compared to other providers (see Table 3). A summary of key BCTs that staff were trained in across providers’ face-to-face courses is shown in Table 4.

**Table 4:** Summary of key BCTs that staff were trained in during face-to-face courses across providers

	Provider A	Provider B	Provider C	Provider D
Behaviour change techniques				
Unique BCTs trained face-to-face	19	34	41	17
Most common BCTs trained	Information about health consequences; social support (unspecified); problem solving	Information about health consequences; goal setting (outcome); goal setting (behaviour)	Information about health consequences; social support (unspecified)	Social support (unspecified); information about health consequences
Common self-regulatory BCTs trained	Problem solving; behaviour substitution; self-monitoring of outcome(s) of behaviour	Goal setting (outcome); goal setting (behaviour); problem solving	Goal setting (behaviour); self-monitoring of behaviour; problem solving	Action planning; feedback on outcome(s) of behaviour; self-monitoring of behaviour
Depth of BCT training				
Most common depth trained	Instructed; practiced; modelled	Instructed; directed	Instructed; directed	Instructed; directed

### Delivery Style of Staff Training

The key characteristics of each providers’ staff training courses is summarised in Table 5. There was variation in the way each provider delivered their staff training. For example, provider A’s training was more self-directed, where trainees role-played sessions described in the manual and received feedback from the trainers. Provider B delivered training which followed an educational format (e.g. use of PowerPoint and trainees taking notes). Provider C’s training was experiential and focused on the delivery of sessions in the manual to trainees with the trainer providing instruction on how each activity should be delivered, and provider D took a more informal approach, in which trainees had input on areas in which they felt they needed more training. Pre-course reading documents also varied between provider, which included journal articles, pre-training handbooks, and reading to supplement the programme manual.

**Table 5:** Summary of key characteristics of provider training in the NHS-DPP

	Provider A	Provider B	Provider C	Provider D
Training delivery				
Pre-course reading	Yes	Yes	Yes	Yes
Length of training	3 days	5 days	3 days	2 days
Delivery style	Role-play and feedback	Educational	Experiential and group discussions	Trainee-led and group discussions
Other skills trained	Facilitation behaviours; group management	Initial assessments; blood glucose testing	Blood glucose testing	Initial assessments; blood glucose testing; group management

All providers encouraged trainees to role-play some aspects of delivery, but some providers had more emphasis on this than others did. Two providers had a particular emphasis on training group facilitation behaviours (e.g. open listening, empathising and group management).

## Discussion

Overall, providers trained staff in 66% of BCTs present in their NHS-DPP intervention plans. The current research team’s previous fidelity evaluation comparing BCTs specified in providers’ intervention plans to BCTs specified in the evidence base showed that providers planned to deliver 74% of BCTs [26]. Thus, a drift in fidelity from the NHS-DPP design to the training is evident. Fidelity was notably higher for two providers in comparison to the other two providers who only trained staff in approximately half of BCTs they had planned to deliver.

Despite variation across providers in their training delivery style, all four providers did train staff in 11 common BCTs, the majority of which were self-regulatory. That is, BCTs designed to help individuals to take control of their behaviour such as goal setting, self-monitoring and problem solving. Such BCTs have the strongest evidence for effectiveness in behaviour change [10], and the evidence review underpinning the NHS-DPP stated these BCTs should be embedded in the programme [25]. It is encouraging that all four providers trained their staff in these BCTs. However, three of the self-regulatory BCTs were only directed to be delivered without demonstration to trainees or the opportunity for them to practice delivering the BCT themselves, which in turn would increase their capability of delivering these techniques [24].

### What This Study Adds to the Literature

To our knowledge, this study is the first thorough examination of fidelity of staff training of BCTs to the intervention design for any diabetes prevention programme in the world. The most comparable research in terms of staff training of behaviour change interventions is research from the English Stop Smoking Service, which found that the face-to-face skills training course for stop smoking practitioners appeared to increase trainees’ confidence in delivering smoking cessation support, including the delivery of BCTs for behavioural support [33]. Further, research found that service users in the Stop Smoking Service were more likely to have quit smoking if their practitioner had completed the relevant staff training, thus highlighting the importance of evidence-based training for staff delivering behaviour change programmes [34].

The only other study to date that has applied the BCTTv1 [9] to staff training courses is research which evaluated the use of BCTs in continued professional development courses for medical staff [35]. In this study, researchers coded BCTs *delivered* in training courses to change health professional practice behaviours [35]. However, the current study assessed whether NHS-DPP trainee facilitators were *trained* in the delivery of BCTs to participants attending diabetes prevention group sessions.

### Implications for Practice

Results from the current study highlighted that fidelity of BCTs in the NHS-DPP staff training to the intervention plans was 66% across the four providers, though two providers only trained staff in approximately half of the BCTs in their intervention plans. Providers should review their training to ensure staff are trained in all key components of their planned intervention designs; if the training does not include the key behaviour change components, then it is likely that these key components will also be missing in the delivery of the intervention [15]. When interpreting effectiveness of the NHS-DPP, it must be taken into account that training varies across providers and staff may be trained in only half of the planned BCTs. The findings reported here will be useful when considering the lack of fidelity of delivery of the programme that our subsequent research has identified [36, 37], in terms of whether deficiencies in training appear to impact on delivery across providers.

Our observations suggested that the most common way in which providers trained their staff was by instructing them how to deliver BCTs, sometimes without demonstration of how to deliver these BCTs in group settings or the practice of BCT delivery. The importance of role-play in staff training has been emphasised as a way to assess skill acquisition [12]. For example, previous research demonstrated that training for a walking intervention that involved role-play with feedback and competency assessments resulted in 80% fidelity of delivery in primary care; this was high fidelity in comparison to previous interventions [38]. Further, a systematic review and meta-analysis found that high-quality staff training improved health outcomes in behaviour change interventions, especially in training that included a combination of educational and practical activities, rather than educational components alone [39]. Thus, providers could further review their training to ensure that staff are trained sufficiently thoroughly so that trainees are clear on how exactly to deliver particular BCTs for different activities. Providers should therefore allocate enough training days or sessions to deliver comprehensive training of the use of BCTs specifically, especially as trainees may come from a range of backgrounds with varying experience in the delivery of BCTs.

### Implications for Research

This study is the first known assessment of BCT content and the first thorough fidelity evaluation of a national diabetes prevention programme in the world. The paper extends on previous fidelity research which to date has focused more on evaluating fidelity of intervention delivery [14]. The author-developed framework for assessing the depth of BCT training could be used in future evaluations to determine the comprehensiveness of BCT training delivered to staff and may help to identify gaps in behaviour change content which could be trained more thoroughly.

The current evaluation did highlight that providers who trained their staff in fewer BCTs, and subsequently had a lower fidelity of trained BCTs to intervention plans, did train their staff in a higher proportion of BCTs in more depth (e.g. role playing BCT delivery rather than just instructing staff to deliver a BCT) compared to providers who demonstrated higher fidelity. Future research could assess whether the depth of BCT training has an impact on: (a) the fidelity of delivery of BCTs in the field, and (b) the overall effectiveness of the intervention, especially for self-regulatory BCTs in which there is the most evidence for their effectiveness in changing health behaviours [10]. Such research may establish whether providing in-depth training on how to deliver BCTs (such as demonstration and practice) would increase the actual delivery of BCTs in the field, and whether this subsequently has an impact on the overall effectiveness of a programme.

In addition to the assessment of trained BCTs, the training of facilitation skills such as active listening, empathising and group management are also important for effective delivery of group intervention sessions [40], which may have a subsequent impact on group rapport and retention of service users on the programme. Training in facilitation skills was observed across provider training in the NHS-DPP, though some providers placed more emphasis on this than others did. It was beyond the scope of the current evaluation to assess fidelity of staff competencies other than the behaviour change content of the NHS-DPP. However, future research could further assess fidelity of trained group facilitation behaviours and the impact this has on the delivery and outcomes of an intervention.

### Strengths and Limitations

This fidelity analysis used a standardised BCT framework [9] and obtained all relevant documentation (e.g. pre-course reading materials of all mandatory staff training) to complete the analysis. The use of audio recordings to capture staff training content is considered a ‘gold standard’ for fidelity evaluations [12], and authors have demonstrated that it is a reliable method for assessing fidelity of BCTs in staff training as external evaluators. This study is one of the first fidelity evaluations of a national programme, and to the authors’ knowledge, one of the only studies to assess fidelity of the staff training to the intervention design with a focus on behaviour change content. Further, researchers developed a coding framework to assess the depth in which staff were trained in BCTs; to our knowledge, this is the first study to assess the depth of BCT training.

Despite the merits of the current study, researchers were only able to observe one set of core training courses for each provider. Authors do not know the extent to which the same results would have been obtained if a different set of training courses were observed. The staff training for each provider was observed in four different geographical locations across England, obtaining as diverse and varied of sample of training as was feasible. However, authors cannot be sure whether providers selected training courses and sites based on what they thought would represent their best training courses; if this is the case, there may be a bias towards observing the ‘better’ training courses.

Further, authors did not observe any ‘top-up’ training courses or other forms of continued professional development due to the time and resources required for intensive observation and the variation in the types of further training courses that were delivered across providers. However, the core training courses observed across providers were mandatory training in order for NHS-DPP facilitators to deliver the programme in the field, and NHS-DPP facilitators were delivering group sessions on the basis of this core training alone, therefore offering the best representation of training that all staff across the NHS-DPP must have received.

## Conclusions

This fidelity analysis found that overall providers trained their staff in 66% of the BCTs present in their intervention plans. The research team’s previous document analysis of the NHS-DPP design, which compared BCTs specified in providers’ intervention plans to the BCTs specified in the underlying evidence base, yielded 74% fidelity of BCTs [26]. Thus, a drift in fidelity from the intervention design to the training stage is evident, and may result in a further dilution in fidelity of the delivery of BCTs in the NHS-DPP. Given that BCTs are the ‘active ingredients’ that can produce behaviour change in individuals, it is vital that staff are adequately trained in how to deliver these techniques in group settings encouraging lifestyle behaviour change. Further, our results suggest that providers may need to incorporate more comprehensive BCT training into their core training courses to ensure that trainee staff are not only told which BCTs should be delivered in the NHS-DPP, but shown how to deliver these BCTs for various group activities and given the opportunity to practice BCT delivery during their training courses.

## Supplementary Information

Below is the link to the electronic supplementary material.

Supplementary file1 (DOCX 38 KB)
