# Supplementary material for: The Fidelity of Training in Behaviour Change Techniques to Intervention Design in a National Diabetes Prevention Programme
Source: Int J Behav Med. 2021 Feb 9;28(6):671–82. doi: 10.1007/s12529-021-09961-5 (PMC8551141; doi:10.1007/s12529-021-09961-5)
Supplement: Supplementary file 1 — Supplementary file1 (DOCX 38 KB) [file 12529_2021_9961_MOESM1_ESM.docx]

**Electronic Supplementary Material 1: Staff Training Observations Data Collection Form**

**Observation of DPP Staff Training
Data Collection Form**

**Researcher: ……………………………………………………………………………………………**

**Date of observation: ……………………………………………………………………………………………**

**Provider organisation: ……………………………………………………………………………………………**

**Location and venue of training: ……………………………………………………………………………………………**

**Number of trainers: ……………………………………………………………………………………………**

**Information about trainer(s) (background, time in post, experience delivering DPP, m/f): ……………………………………………………………………………………………………………………………………………………………………………………….……………………………………………………………………………………………………………………………………………………………………………………….………………………………………………………………………………………**

**Number of trainees: ……………………………………………………………………………………………**

**Information about trainees (background, time in post, experience delivering DPP, m/f): ………………………………………………………………………………………………………………………………………………………………………………………………………………………………………………………………………………………………………………………………………………………………………………………………………………………………………………………………………**

**Session number: …………………………………………………………………………………………..**

**Time (start/end): ……………………………………………………………………………………………**

**Duration of session: ……………………………………………………………………………………………**

**Recorder: ……………………………………………………………………………………………**

**File number on recorder: …………………………………………………………………………………………..**

**Materials used: …………………………………………………………………………………………………………………………...... ………………………………………………………………………………………………………………………………………………………..……………………………………………………………………………………………………………………………………………………………………………………………………………………………………………………………………………………………………………….**

**Observation instructions**

1. Use a new coding sheet for each session of the training (a session ends when there is a break for e.g. coffee, lunch, end of the day, and a new one starts following this break). Stop the audio-recording during breaks and start a new recording for each new session. Note the audio-recording number for each session.
2. Position the audio-recorder either on an unobstructed flat surface near the trainer (e.g. table at the front of the room), or ask the trainer to wear the audio-recorder on their belt with a tie-clip microphone attached.
3. Start the audio-recorder and stopwatch at the same time so that time-stamps can be taken from the stopwatch while the recorder captures sound at the front of the room.
4. **Materials:** Keep a note of any materials used to deliver the training (e.g. handouts, flipchart, slides, visual aids) in the section above.
5. **Activity:** Keep a note of each topic of discussion or activity carried out during the training in the order they occur (e.g. group discussion, information about the course, instructions how to deliver a delivery activity, knowledge quiz) and the subject of each activity (e.g. diet, exercise, stress).
6. **Theory:** Keep a note of any theory explicitly mentioned to trainees, or any reference to mechanisms of actions (i.e. the constructs or predictors of behaviour targeted by BCTs in order to change service user behaviour, e.g. self-efficacy, social norms)
7. **BCTs:** Keep a note of any behaviour change techniques the trainees are ***trained to deliver*** to patients during the DPP delivery sessions. Use labels from the BCT taxonomy v1.

- Specify if the BCTs are generated by the trainer or the trainees/participants during the session.

1. **Comments:** Make a note of any problems, uncertainties, unusual occurrences, etc.

If apparent, can note here confidence in presence of BCT, whether the BCT is optional or essential, target behaviour of the BCT, and suggested mode of delivery to service users, but these will largely be captured by later coding of transcripts.

| **Time** | **Activity/ topic of discussion** | **Theory/ mechanisms of action** | **Behaviour Change Technique**  Label from BCTTv1.  (Specify if BCT was generated by the participants or the trainer) | **Comments** |
| --- | --- | --- | --- | --- |
|  |  |  |  |  |
|  |  |  |  |  |

**Electronic Supplementary Material 2: Behaviour Change Technique Coding Procedures**

Behaviour change techniques (BCTs) were coded using an author-developed table, which included the following columns:

- BCT name
- Confidence of presence
- Information about behavioural targets (e.g. diet, physical activity)
- Deliverer of BCT (e.g. trainer, participant, group)
- Depth of trained BCT (e.g. informed about, directed to, instructed how, demonstrated how, practiced how, modelled how)
- Whether the BCT was optional or required of the NHS-DPP deliverers
- Location in the audio recording in which BCT was trained (minutes, seconds)
- Summarised evidence of the trained BCT

Coding rules stated that new BCTs would be coded when staff were trained on the commencement of a new activity or if a different health behaviour (e.g. diet, physical activity) was targeted. The level of target behaviour was also documented when coding the BCT ‘information about health consequences’ (e.g., levels of the target behaviour ‘diet’ included information about carbohydrates, fats, sugar, etc.) as the authors felt these were distinct pieces of information targeting distinct behaviours.

BCTs present in both providers’ intervention plans and providers’ staff training were documented as indicating fidelity to the intervention design. BCTs stated in providers’ intervention plans that were not present in their staff training, and additional BCTs identified in each providers’ staff training which were not otherwise specified, were documented as indicating non-fidelity to the intervention design.

**Electronic Supplementary Material 3: Kappa Values**

**Table S1. Kappa Values for NHS-DPP Intervention Design Documents**

| Source Document | Kappa Value |
| --- | --- |
| NHS Service Specification | 0.84 |
| NICE PH38 guidelines | 0.87 |
| Provider 1 | |
| Framework response | 0.76 |
| Programme manual | 0.81 |
| Provider 2 | |
| Framework response | 0.88 |
| Programme manual | 0.75 |
| Provider 3 | |
| Framework response | 0.84 |
| Programme manual | 0.79 |
| Provider 4 | |
| Framework response | 0.75 |
| Programme manual | 0.76 |

*Note. Providers were labelled 1, 2, 3, 4 in Hawkes et al. (2020) to preserve provider anonymity. Providers 1, 2, 3 and 4 do not correspond to providers A, B, C and D.*

**Table S2. Kappa Values for Sample of Providers’ Staff Training Sessions**

| Source Document | Kappa Value |
| --- | --- |
| Provider A | |
| Training session 1 | 0.65 |
| Provider B | |
| Training session 1 | 0.80 |
| Training session 2 | 0.61 |
| Provider C | |
| Training session 1 | 0.68 |
| Provider D | |
| Training session 1 | 0.80 |

*Note. Providers A, B, C and D do not correspond to providers 1, 2, 3 and 4.*

**Electronic Supplementary Material 4: Behaviour Change Technique Definitions**

**Table S3. Behaviour Change Technique Definitions**

| **Behaviour Change Technique** | **Definition** |
| --- | --- |
| Goal setting for health behaviours [1.1] | Set or agree on a goal defined in terms of the behaviour to be achieved. |
| Problem solving [1.2] | Prompt the person to analyse factors influencing the behaviour and generate or select strategies that include overcoming barriers or increasing facilitators. |
| Goal setting for health outcomes [1.3] | Set or agree on a goal defined in terms of a positive outcome of wanted behaviour. |
| Action planning [1.4] | Prompt detailed planning of the performance of the behaviour (must include at least one of context, frequency, duration and intensity). |
| Reviewing behaviour goals [1.5] | Review behaviour goal(s) jointly with the person and consider modifying goal(s) or behaviour change strategy in light of achievement. |
| Discrepancy between current behaviour and goal [1.6] | Draw attention to discrepancies between a person’s current behaviour (in terms of the *form, frequency, duration, or intensity* of that behaviour) and the person’s previously set outcome goals, behavioural goals or action plans. |
| Reviewing outcome goals [1.7] | Review outcome goal(s) jointly with the person and consider modifying goal(s) in light of achievement. |
| Commitment [1.9] | Ask the person to affirm or reaffirm statements indicating commitment to change the behaviour. |
| Giving feedback on behaviour [2.2] | Monitor and provide informative or evaluative feedback on performance of the behaviour. |
| Self-monitoring of behaviour [2.3] | Establish a method for the person to monitor and record their behaviour(s) as part of a behaviour change strategy. |
| Self-monitoring of outcomes of behaviour [2.4] | Establish a method for the person to monitor and record the outcome(s) their behaviour as part of a behaviour change strategy. |
| Monitoring outcome of behaviour by others without feedback [2.5] | Observe or record outcomes of behaviour with the person’s knowledge as part of a behaviour change strategy. |
| Biofeedback [2.6] | Provide feedback about the body *(e.g. physiological or biochemical state)* using an external monitoring device as part of a behaviour change strategy. |
| Giving feedback on outcomes of behaviour [2.7] | Monitor and provide feedback on the outcome of performance of the behaviour. |
| Unspecified social support [3.1] | Advise on, arrange or provide social support or non-contingent praise or reward for performance of the behaviour. |
| Practical social support [3.2] | Advise on, arrange or provide practical help for performance of the behaviour. |
| Emotional social support [3.3] | Advise on, arrange or provide emotional social support for performance of the behaviour. |
| Instruction on how to perform a behaviour [4.1] | Advise or agree on how to perform the behaviour (includes ‘Skills training’). |
| Information about antecedents [4.2] | Provide information about antecedents (*e.g. social and environmental situations and events, emotions, cognitions)* that reliably predict performance of the behaviour. |
| Information about health consequences [5.1] | Provide information about health consequences of performing the behaviour. |
| Salience of consequences [5.2] | Use methods specifically designed to emphasise the consequences of performing the behaviour with the aim of making them more memorable (goes beyond informing about consequences). |
| Salience of behaviours ^a^ | Use methods specifically designed to emphasise the behaviour when linking the behaviour to the consequence with the aim of making the consequence more memorable. |
| Giving information about social and environmental consequences [5.3] | Provide information (e.g. written, verbal, visual) about social and environmental consequences of performing the behaviour. |
| Anticipated regret [5.5] | Induce or raise awareness of expectations of future regret about performance of the unwanted behaviour. |
| Giving information about emotional consequences [5.6] | Provide information (e.g. written, verbal, visual) about emotional consequences of performing the behaviour. |
| Giving a demonstration of the behaviour [6.1] | Provide an observable sample of the performance of the behaviour, directly in person or indirectly e.g. via film, pictures, for the person to aspire to or imitate. |
| Social comparison [6.2] | Draw attention to others’ performance to allow comparison with the person’s own performance. |
| Prompts/cues [7.1] | Introduce or define environmental or social stimulus with the purpose of prompting or cueing the behaviour. |
| Remove access to the reward [7.4] | Advise or arrange for the person to be separated from situations in which unwanted behaviour can be rewarded in order to reduce the behaviour. |
| Behavioural practice [8.1] | Prompt practice or rehearsal of the performance of the behaviour in order to increase habit or skill. |
| Behaviour substitution [8.2] | Prompt the substitution of the unwanted behaviour with a wanted or neutral behaviour. |
| Habit formation [8.3] | Prompt rehearsal and repetition of the behaviour in the same context repeatedly so that the context elicits the behaviour. |
| Habit reversal [8.4] | Prompt rehearsal and repetition of an alternative behaviour to replace an unwanted habitual behaviour. |
| Overcorrection [8.5] | Ask to repeat the wanted behaviour in an exaggerated way following an unwanted behaviour. |
| Graded tasks [8.7] | Set easy-to-perform tasks, making them increasingly difficult, but achievable, until behaviour is performed. |
| Credible source [9.1] | Present verbal or visual communication from a credible source in favour of or against the behaviour. |
| Pros and cons [9.2] | Advise the person to identify and compare reasons for wanting (pros) and not wanting to (cons) change the behaviour. |
| Comparative imagining of future outcomes [9.3] | Prompt or advise the imagining and comparing of future outcomes of changed versus unchanged behaviour. |
| Material incentive (behaviour) [10.1] | Inform that money, vouchers or other valued objects will be delivered if and only if there has been effort and/or progress in performing the behaviour. |
| Material reward (behaviour) [10.2] | Arrange for the delivery of money, vouchers or other valued objects if and only if there has been effort and/or progress in performing the behaviour. |
| Non-specific reward [10.3] | Arrange delivery of a reward if and only if there has been effort and/or progress in performing the behaviour. |
| Social reward [10.4] | Arrange verbal or non-verbal reward if and only if there has been effort and/or progress in performing the behaviour. |
| Social incentive [10.5] | Inform that a verbal or non-verbal reward will be delivered if and only if there has been effort and/or progress in performing the behaviour. |
| Non-specific incentive [10.6] | Inform that a reward will be delivered if and only if there has been effort and/or progress in performing the behaviour. |
| Self-incentive [10.7] | Plan to reward self in future if and only if there has been effort and/or progress in performing the behaviour. |
| Incentive (outcome) [10.8] | Inform that a reward will be delivered if and only if there has been effort and/or progress in achieving the behavioural outcome. |
| Self-reward [10.9] | Prompt self-praise or self-reward if and only if there has been effort and/or progress in performing the behaviour. |
| Reward (outcome) [10.10] | Arrange for the delivery of a reward if and only if there has been effort and/or progress in achieving the behavioural outcome. |
| Pharmacological support [11.1] | Provide, or encourage the use of or adherence to, drugs to facilitate behaviour change. |
| Reduce negative emotions [11.2] | Advise on ways of reducing negative emotions to facilitate performance of the behaviour. |
| Increase positive emotions ^b^ | Advise on ways of increasing positive emotions to facilitate performance of the behaviour. |
| Restructuring the physical environment [12.1] | Change, or advise to change the physical environment in order to facilitate performance of the wanted behaviour or create barriers to the unwanted behaviour. |
| Restructuring the social environment [12.2] | Change, or advise to change the social environment in order to facilitate performance of the wanted behaviour or create barriers to the unwanted behaviour. |
| Avoiding/reducing exposure to cues for the behaviour [12.3] | Advise on how to avoid exposure to specific social and contextual/physical cues for the behaviour, including changing daily or weekly routines. |
| Distraction [12.4] | Advise or arrange to use an alternative focus for attention to avoid triggers for unwanted behaviour. |
| Adding objects to the environment [12.5] | Add objects to the environment in order to facilitate performance of the behaviour. |
| Identification of self as role model [13.1] | Inform that one's own behaviour may be an example to others. |
| Framing/reframing [13.2] | Suggest the deliberate adoption of a perspective or new perspective on behaviour (e.g. its purpose) in order to change cognitions or emotions about performing the behaviour. |
| Verbal persuasion about capability [15.1] | Tell the person that they can successfully perform the wanted behaviour, arguing against self-doubts and asserting that they can and will succeed. |
| Mental rehearsal of successful performance [15.2] | Advise to practise imagining performing the behaviour successfully in relevant contexts. |
| Focus on past success [15.3] | Advise to think about or list previous successes in performing the behaviour (or parts of it). |
| Self-talk [15.4] | Prompt positive self-talk (aloud or silently) before and during the behaviour. |

*Note: Definitions are summarised from BCTTv1. Numbers in square brackets are corresponding number in BCTTv1.*

*^a^ Salience of behaviours was not listed in the BCTTv1, but has been identified as a new behaviour change technique by the authors of this paper.*

*^b^ Increase positive emotions is not listed in the BCTTv1, but was noted by the authors for inclusion in the next version of the taxonomy.*

**Electronic Supplementary Material 5: Sensitivity Analyses**

**Table S4. BCTs Present in Staff Training Compared to Each Providers’ Framework Responses**

|  | No. of BCTs planned in framework response | No. (%) of planned BCTs included in staff training | | No. of planned BCTs not included in training | No. of unplanned BCTs included in staff training |
| --- | --- | --- | --- | --- | --- |
|  |  | *n* | % |  |  |
| Provider A | 32 | 14 | 43.8 | 18 | 5 |
| Provider B | 24 | 22 | 91.7 | 2 | 14 |
| Provider C | 27 | 24 | 88.9 | 3 | 20 |
| Provider D | 32 | 18 | 56.3 | 14 | 4 |

*Note. BCTs in staff training include those present in the face-to-face training and pre-course reading materials.*

**Table S5. BCTs Present in Staff Training Compared to Each Providers’ Programme Manuals**

|  | No. of BCTs planned in programme manuals | No. (%) of planned BCTs included in staff training | | No. of planned BCTs not included in training | No. of unplanned BCTs included in staff training |
| --- | --- | --- | --- | --- | --- |
|  |  | *n* | % |  |  |
| Provider A | 28 | 17 | 60.7 | 11 | 2 |
| Provider B | 35 | 29 | 82.9 | 6 | 7 |
| Provider C | 45 | 38 | 84.4 | 7 | 6 |
| Provider D | 23 | 14 | 60.9 | 9 | 8 |

*Note. BCTs in staff training include those present in the face-to-face training and pre-course reading materials.*

**Table S6. BCTs Present in Staff Training Compared to Each Providers’ Programme Manuals, Including Initial Assessment Protocols**

|  | No. of BCTs planned in programme manuals | No. (%) of planned BCTs included in staff training | | No. of planned BCTs not included in training | No. of unplanned BCTs included in staff training |
| --- | --- | --- | --- | --- | --- |
|  |  | *n* | % |  |  |
| Provider A | 29 | 17 | 58.6 | 12 | 2 |
| Provider B | 37 | 30 | 81.1 | 7 | 6 |
| Provider C | 45 | 38 | 84.4 | 7 | 6 |
| Provider D | 26 | 17 | 65.4 | 9 | 5 |

*Note. BCTs in staff training include those present in the face-to-face training and pre-course reading materials.*

**Electronic Supplementary Material 6: Breakdown of Behaviours Targeted by Each BCT and Depth of BCTs Trained Across Providers in the Face-to-Face Sessions**

**Table S7. Behaviours Targeted and Depth of BCTs Trained in Face-to-Face Training of Provider A**

| **Behaviour change technique** | **Depth of training** | **Diet** | **PA** | **Alcohol** | **Smoking** | **Unspecified** | **Other** |
| --- | --- | --- | --- | --- | --- | --- | --- |
| Action planning [1.4] | Directed | 0 | 0 | 0 | 0 | 3 | 0 |
| Adding objects to the environment [12.5] | Instructed | 0 | 1 | 0 | 0 | 0 | 0 |
| Avoidance/reducing exposure to cues for the behaviour [12.3] | Directed | 0 | 0 | 0 | 0 | 1 | 0 |
| Behavioural practice/rehearsal [8.1] | Instructed | 0 | 1 | 0 | 0 | 0 | 0 |
| Behaviour substitution [8.2] | Instructed | 1 | 0 | 0 | 0 | 0 | 0 |
| Behaviour substitution [8.2] | Practiced/ modelled | 4 | 1 | 0 | 0 | 0 | 0 |
| Demonstration of the behaviour [6.1] | Instructed | 0 | 1 | 0 | 0 | 0 | 0 |
| Feedback on behaviour [2.6] | Practiced/ modelled | 1 | 1 | 0 | 0 | 0 | 0 |
| Goal setting (behaviour) [1.1] | Instructed | 0 | 1 | 0 | 0 | 0 | 0 |
| Goal setting (outcome) [1.3] | Practiced/ modelled | 0 | 0 | 0 | 0 | 1 | 0 |
| Information about antecedents [4.1] | Instructed | 1 | 0 | 0 | 0 | 0 | 0 |
| Information about antecedents [4.1] | Practiced/ modelled | 1 | 0 | 0 | 0 | 0 | 0 |
| Information about emotional consequences [5.6] | Instructed | 1 | 0 | 0 | 0 | 0 | 0 |
| Information about emotional consequences [5.6] | Practiced/ modelled | 0 | 1 | 0 | 0 | 0 | 0 |
| Information about health consequences [5.1] | Instructed | 2 | 2 | 0 | 0 | 0 | 0 |
| Information about health consequences [5.1] | Practiced/ modelled | 12 | 6 | 0 | 2 | 0 | 0 |
| Instruction on how to perform the behaviour [4.1] | Instructed | 0 | 1 | 0 | 0 | 0 | 0 |
| Problem solving [1.2] | Informed | 0 | 0 | 0 | 0 | 1 | 0 |
| Problem solving [1.2] | Instructed | 0 | 0 | 0 | 0 | 3 | 0 |
| Problem solving [1.2] | Demonstrated | 0 | 0 | 0 | 0 | 1 | 0 |
| Problem solving [1.2] | Practiced/ modelled | 0 | 1 | 0 | 0 | 1 | 0 |
| Prompts/cues [7.1] | Directed | 0 | 0 | 0 | 0 | 1 | 0 |
| Salience of behaviours [x] *^a^* | Practiced/ modelled | 2 | 1 | 0 | 0 | 0 | 0 |
| Self-monitoring of behaviour [2.3] | Practiced/ modelled | 1 | 2 | 0 | 0 | 0 | 0 |
| Self-monitoring of outcome(s) of behaviours [2.4] | Directed | 0 | 0 | 0 | 0 | 1 | 0 |
| Self-monitoring of outcome(s) of behaviours [2.4] | Practiced/ modelled | 0 | 0 | 0 | 0 | 5 | 0 |
| Social support (unspecified) [3.1] | Directed | 0 | 0 | 0 | 0 | 1 | 0 |
| Social support (unspecified) [3.1] | Informed | 0 | 0 | 0 | 0 | 1 | 0 |
| Social support (unspecified) [3.1] | Instructed | 0 | 0 | 0 | 0 | 4 | 0 |

*Note. Numbers in square brackets are corresponding number in BCTTv1. BCTs which were trained once but via multiple depths of training are shown across one row (e.g. ‘practiced/ modelled’)*

*^a^ Salience of behaviours was not listed in the BCTTv1, but has been identified as a new behaviour change technique by the authors of this paper.*

**Table S8. Behaviours Targeted and Depth of BCTs Trained in Face-to-Face Training of Provider B**

| **Behaviour change technique** | **Depth of training** | **Diet** | **PA** | **Alcohol** | **Smoking** | **Unspecified** | **Other** |
| --- | --- | --- | --- | --- | --- | --- | --- |
| Action planning [1.4] | Instructed | 1 | 1 | 0 | 0 | 2 | 0 |
| Action planning [1.4] | Demonstrated | 0 | 0 | 0 | 0 | 1 | 0 |
| Action planning [1.4] | Demonstrated/ modelled | 0 | 1 | 0 | 0 | 1 | 0 |
| Avoidance/reducing exposure to cues for the behaviour [12.3] | Informed about | 0 | 0 | 0 | 0 | 1 | 0 |
| Avoidance/reducing exposure to cues for the behaviour [12.3] | Directed | 1 | 0 | 0 | 0 | 0 | 0 |
| Behavioural practice/rehearsal [8.1] | Demonstrated | 0 | 1 | 0 | 0 | 0 | 0 |
| Behavioural practice/rehearsal [8.1] | Instructed | 1 | 0 | 0 | 0 | 0 | 0 |
| Behaviour substitution [8.2] | Directed | 1 | 0 | 0 | 0 | 0 | 0 |
| Behaviour substitution [8.2] | Instructed | 2 | 1 | 1 | 0 | 0 | 0 |
| Behaviour substitution [8.2] |  | 0 | 0 | 0 | 0 | 0 | 0 |
| Biofeedback [2.6] | Directed | 0 | 0 | 0 | 0 | 2 | 0 |
| Biofeedback [2.6] | Demonstrated/ modelled | 0 | 0 | 0 | 0 | 1 | 0 |
| Credible source | Instructed | 0 | 1 | 0 | 0 | 0 | 0 |
| Commitment [1.9] | Directed | 0 | 0 | 0 | 0 | 3 | 0 |
| Demonstration of behaviour [6.1] | Demonstrated | 0 | 1 | 0 | 0 | 0 | 0 |
| Feedback on behaviour [2.2] | Instructed | 1 | 0 | 0 | 0 | 0 | 0 |
| Feedback on behaviour [2.2] | Demonstrated | 1 | 0 | 0 | 0 | 0 | 0 |
| Feedback on outcome(s) of behaviour [2.7] | Directed | 0 | 0 | 0 | 0 | 4 | 0 |
| Feedback on outcome(s) of behaviour [2.7] | Instructed | 0 | 0 | 0 | 0 | 2 | 0 |
| Feedback on outcome(s) of behaviour [2.7] | Demonstrated | 0 | 0 | 0 | 0 | 1 | 0 |
| Feedback on outcome(s) of behaviour [2.7] | Modelled | 0 | 0 | 0 | 0 | 1 | 0 |
| Focus on past success [15.3] | Instructed | 0 | 0 | 0 | 0 | 3 | 0 |
| Framing/reframing [13.2] | Instructed | 0 | 0 | 0 | 0 | 1 | 0 |
| Graded tasks [8.7] | Directed | 0 | 0 | 0 | 0 | 2 | 0 |
| Goal setting (behaviour) [1.1] | Informed about | 0 | 0 | 0 | 0 | 1 | 0 |
| Goal setting (behaviour) [1.1] | Directed | 4 | 1 | 0 | 0 | 0 | 0 |
| Goal setting (behaviour) [1.1] | Instructed | 1 | 3 | 0 | 0 | 4 | 0 |
| Goal setting (behaviour) [1.1] | Demonstrated | 1 | 0 | 0 | 0 | 1 | 0 |
| Goal setting (behaviour) [1.1] | Demonstrated/ modelled | 0 | 1 | 0 | 0 | 0 | 0 |
| Goal setting (outcome) [1.3] | Informed about | 0 | 0 | 0 | 0 | 1 | 0 |
| Goal setting (outcome) [1.3] | Directed | 0 | 0 | 0 | 0 | 16 | 0 |
| Goal setting (outcome) [1.3] | Demonstrated/ modelled | 0 | 0 | 0 | 0 | 1 | 0 |
| Increase positive emotions [x] *^a^* | Directed | 0 | 0 | 0 | 0 | 1 | 0 |
| Increase positive emotions [x] *^a^* | Instructed | 0 | 0 | 0 | 0 | 1 | 0 |
| Information about antecedents [4.1] | Instructed | 1 | 0 | 0 | 0 | 0 | 0 |
| Information about antecedents [4.1] | Demonstrated | 1 | 0 | 0 | 0 | 0 | 0 |
| Information about emotional consequences [5.6] | Directed | 0 | 1 | 0 | 0 | 0 | 0 |
| Information about health consequences [5.1] | Informed about | 14 | 0 | 0 | 0 | 0 | 0 |
| Information about health consequences [5.1] | Directed | 2 | 1 | 0 | 0 | 0 | 0 |
| Information about health consequences [5.1] | Instructed | 28 | 8 | 0 | 0 | 0 | 4 |
| Information about health consequences [5.1] | Demonstrated | 11 | 0 | 0 | 0 | 0 | 0 |
| Information about health consequences [5.1] | Practiced | 2 | 0 | 0 | 0 | 0 | 0 |
| Information about health consequences [5.1] | Demonstrated/ modelled | 2 | 0 | 1 | 0 | 0 | 0 |
| Instruction on how to perform the behaviour [4.1] | Instructed | 1 | 0 | 0 | 0 | 0 | 0 |
| Instruction on how to perform the behaviour [4.1] | Demonstrated | 0 | 1 | 0 | 0 | 0 | 0 |
| Mental rehearsal of successful performance [15.2] | Instructed | 0 | 0 | 0 | 0 | 1 | 0 |
| Problem solving [1.2] | Directed | 1 | 1 | 0 | 0 | 3 | 0 |
| Problem solving [1.2] | Instructed | 1 | 0 | 0 | 0 | 4 | 1 |
| Problem solving [1.2] | Demonstrated | 0 | 0 | 0 | 0 | 1 | 0 |
| Pros and cons [9.2] | Directed | 0 | 0 | 0 | 0 | 1 | 0 |
| Reduce negative emotions [11.2] | Directed | 0 | 0 | 0 | 0 | 1 | 0 |
| Reduce negative emotions [11.2] | Demonstrated/ modelled | 0 | 0 | 0 | 0 | 1 | 0 |
| Restructuring the physical environment [12.1] | Directed | 1 | 0 | 0 | 0 | 0 | 0 |
| Review behaviour goals [1.5] | Directed | 2 | 0 | 0 | 0 | 0 | 0 |
| Review behaviour goals [1.5] | Instructed | 1 | 0 | 0 | 0 | 0 | 0 |
| Review outcome goals [1.7] | Directed | 0 | 0 | 0 | 0 | 7 | 0 |
| Review outcome goals [1.7] | Instructed | 0 | 0 | 0 | 0 | 4 | 0 |
| Salience of consequences [5.2] | Instructed | 1 | 0 | 0 | 0 | 1 | 0 |
| Self-monitoring of behaviour [2.3] | Informed about | 0 | 0 | 1 | 0 | 0 | 0 |
| Self-monitoring of outcome(s) of behaviours [2.4] | Directed | 0 | 0 | 0 | 0 | 3 | 0 |
| Self-monitoring of outcome(s) of behaviours [2.4] | Instructed | 0 | 0 | 0 | 0 | 1 | 0 |
| Self-reward [10.9] | Instructed | 0 | 0 | 0 | 0 | 1 | 0 |
| Social reward [10.4] | Directed | 0 | 1 | 0 | 0 | 0 | 0 |
| Social reward [10.4] | Instructed | 0 | 0 | 0 | 0 | 1 | 0 |
| Social support (unspecified) [3.1] | Directed | 0 | 1 | 0 | 0 | 2 | 0 |
| Social support (unspecified) [3.1] | Instructed | 0 | 0 | 0 | 0 | 7 | 1 |

*Note. Numbers in square brackets are corresponding number in BCTTv1. BCTs which were trained once but via multiple depths of training are shown across one row (e.g. ‘demonstrated/ modelled’)*

*^a^ Increase positive emotions is not listed in the BCTTv1, but was noted by the authors for inclusion in the next version of the taxonomy.*

**Table S9. Behaviours Targeted and Depth of BCTs Trained in Face-to-Face Training of Provider C**

| **Behaviour change technique** | **Depth of training** | **Diet** | **PA** | **Alcohol** | **Smoking** | **Unspecified** | **Other** |
| --- | --- | --- | --- | --- | --- | --- | --- |
| Action planning [1.4] | Instructed | 5 | 2 | 0 | 0 | 1 | 0 |
| Adding objects to the environment [12.5] | Informed about | 0 | 1 | 0 | 0 | 0 | 0 |
| Adding objects to the environment [12.5] | Directed | 0 | 1 | 0 | 0 | 0 | 0 |
| Adding objects to the environment [12.5] | Instructed | 1 | 0 | 0 | 0 | 0 | 0 |
| Avoidance/reducing exposure to cues for the behaviour [12.3] | Directed | 1 | 0 | 0 | 0 | 0 | 0 |
| Avoidance/reducing exposure to cues for the behaviour [12.3] | Instructed | 5 | 0 | 0 | 0 | 0 | 0 |
| Behavioural practice/rehearsal [8.1] | Demonstrated | 0 | 1 | 0 | 0 | 0 | 0 |
| Behaviour substitution [8.2] | Directed | 2 | 0 | 0 | 0 | 0 | 0 |
| Behaviour substitution [8.2] | Instructed | 12 | 1 | 1 | 0 | 0 | 0 |
| Behaviour substitution [8.2] | Demonstrated/ modelled | 3 | 0 | 0 | 0 | 0 | 0 |
| Biofeedback [2.6] | Directed | 0 | 0 | 0 | 0 | 2 | 0 |
| Commitment [1.9] | Directed | 4 | 0 | 0 | 0 | 1 | 0 |
| Distraction [12.4] | Instructed | 1 | 0 | 0 | 0 | 1 | 0 |
| Feedback on behaviour [2.2] | Instructed | 5 | 3 | 1 | 0 | 0 | 0 |
| Feedback on behaviour [2.2] | Demonstrated/ modelled | 3 | 0 | 0 | 0 | 0 | 0 |
| Feedback on outcome(s) of behaviour [2.7] | Directed | 0 | 0 | 0 | 0 | 3 | 0 |
| Feedback on outcome(s) of behaviour [2.7] | Instructed | 0 | 0 | 0 | 0 | 2 | 0 |
| Framing/reframing [13.2] | Informed about | 0 | 0 | 0 | 0 | 1 | 0 |
| Framing/reframing [13.2] | Instructed | 1 | 0 | 0 | 0 | 0 | 0 |
| Framing/reframing [13.2] | Demonstrated/ modelled | 0 | 0 | 0 | 0 | 1 | 0 |
| Graded tasks [8.7] | Directed | 0 | 1 | 0 | 0 | 1 | 0 |
| Graded tasks [8.7] | Instructed | 3 | 4 | 0 | 0 | 0 | 0 |
| Goal setting (behaviour) [1.1] | Directed | 2 | 3 | 0 | 0 | 3 | 0 |
| Goal setting (behaviour) [1.1] | Instructed | 8 | 7 | 1 | 0 | 0 | 0 |
| Goal setting (outcome) [1.3] | Directed | 0 | 0 | 0 | 0 | 8 | 0 |
| Goal setting (outcome) [1.3] | Instructed | 0 | 0 | 0 | 0 | 4 | 0 |
| Incentive (outcome) [10.8] | Instructed | 0 | 0 | 0 | 0 | 1 | 0 |
| Information about antecedents [4.1] | Informed about | 2 | 0 | 0 | 0 | 0 | 0 |
| Information about antecedents [4.1] | Directed | 1 | 0 | 0 | 0 | 0 | 0 |
| Information about antecedents [4.1] | Instructed | 1 | 0 | 0 | 0 | 0 | 0 |
| Information about antecedents [4.1] | Demonstrated | 1 | 0 | 0 | 0 | 0 | 0 |
| Information about antecedents [4.1] | Modelled | 1 | 0 | 0 | 0 | 1 | 0 |
| Information about emotional consequences [5.6] | Instructed | 1 | 2 | 0 | 0 | 0 | 0 |
| Information about emotional consequences [5.6] | Demonstrated/ modelled | 0 | 1 | 0 | 0 | 0 | 0 |
| Information about health consequences [5.1] | Informed | 0 | 0 | 0 | 0 | 0 | 1 |
| Information about health consequences [5.1] | Instructed | 20 | 10 | 3 | 0 | 0 | 0 |
| Information about health consequences [5.1] | Demonstrated/ modelled | 0 | 8 | 2 | 0 | 0 | 0 |
| Information about health consequences [5.1] | Practiced | 1 | 0 | 0 | 0 | 0 | 0 |
| Information about health consequences [5.1] | Modelled | 1 | 0 | 0 | 0 | 0 | 0 |
| Information about social and environmental consequences [5.3] | Instructed | 0 | 1 | 0 | 0 | 0 | 0 |
| Material incentive (behaviour) [10.1] | Informed about | 0 | 1 | 0 | 0 | 0 | 0 |
| Monitoring of outcome(s) of behaviour without feedback [2.5] | Directed | 0 | 0 | 0 | 0 | 1 | 0 |
| Monitoring of outcome(s) of behaviour without feedback [2.5] | Instructed | 0 | 0 | 0 | 0 | 1 | 0 |
| Non-specific reward [10.3] | Informed about | 0 | 0 | 0 | 0 | 1 | 0 |
| Non-specific reward [10.3] | Directed | 0 | 0 | 0 | 0 | 1 | 0 |
| Problem solving [1.2] | Directed | 2 | 3 | 0 | 0 | 2 | 0 |
| Problem solving [1.2] | Instructed | 3 | 1 | 0 | 0 | 0 | 0 |
| Problem solving [1.2] | Demonstrated/ modelled | 5 | 1 | 1 | 0 | 0 | 0 |
| Prompts/cues [7.1] | Instructed | 0 | 1 | 0 | 0 | 0 | 0 |
| Pros and cons [9.2] | Directed | 1 | 0 | 0 | 0 | 0 | 0 |
| Reduce negative emotions [11.2] | Demonstrated/ modelled | 0 | 0 | 0 | 0 | 2 | 0 |
| Reduce negative emotions [11.2] | Practiced/ modelled | 0 | 0 | 0 | 0 | 1 | 0 |
| Remove access to the reward [7.4] | Instructed | 2 | 0 | 0 | 0 | 0 | 0 |
| Restructuring the social environment [12.2] | Instructed | 0 | 1 | 1 | 0 | 0 | 0 |
| Review behaviour goals [1.5] | Directed | 0 | 0 | 0 | 0 | 1 | 0 |
| Review behaviour goals [1.5] | Instructed | 0 | 0 | 0 | 0 | 1 | 0 |
| Review outcome goals [1.7] | Directed | 0 | 0 | 0 | 0 | 2 | 0 |
| Review outcome goals [1.7] | Instructed | 0 | 0 | 0 | 0 | 2 | 0 |
| Reward (outcome) [10.10] | Directed | 0 | 0 | 0 | 0 | 3 | 0 |
| Reward (outcome) [10.10] | Instructed | 0 | 0 | 0 | 0 | 4 | 0 |
| Salience of behaviours [x] *^b^* | Demonstrated/ modelled | 3 | 1 | 0 | 0 | 0 | 0 |
| Self-monitoring of behaviour [2.3] | Directed | 7 | 7 | 1 | 0 | 0 | 0 |
| Self-monitoring of behaviour [2.3] | Instructed | 5 | 4 | 0 | 0 | 0 | 0 |
| Self-monitoring of outcome(s) of behaviours [2.4] | Directed | 0 | 0 | 0 | 0 | 4 | 0 |
| Self-monitoring of outcome(s) of behaviours [2.4] | Instructed | 0 | 0 | 0 | 0 | 4 | 0 |
| Self-reward [10.9] | Instructed | 0 | 1 | 0 | 0 | 1 | 0 |
| Self-talk [15.4] | Directed | 0 | 0 | 0 | 0 | 1 | 0 |
| Social comparison [6.2] | Instructed | 0 | 2 | 0 | 0 | 0 | 0 |
| Social incentive [10.5] | Informed about | 0 | 0 | 0 | 0 | 1 | 0 |
| Social incentive [10.5] | Instructed | 0 | 1 | 0 | 0 | 0 | 0 |
| Social reward [10.4] | Directed | 0 | 0 | 0 | 0 | 3 | 0 |
| Social reward [10.4] | Instructed | 0 | 3 | 0 | 0 | 3 | 0 |
| Social support (emotional) [3.3] | Instructed | 0 | 0 | 0 | 0 | 1 | 0 |
| Social support (unspecified) [3.1] | Directed | 0 | 0 | 0 | 0 | 3 | 0 |
| Social support (unspecified) [3.1] | Instructed | 5 | 6 | 0 | 0 | 10 | 0 |

*Note. Numbers in square brackets are corresponding number in BCTTv1. BCTs which were trained once but via multiple depths of training are shown across one row (e.g. ‘demonstrated/ modelled’)*

*^a^ Increase positive emotions is not listed in the BCTTv1, but was noted by the authors for inclusion in the next version of the taxonomy.*

*^b^ Salience of behaviours was not listed in the BCTTv1, but has been identified as a new behaviour change technique by the authors of this paper.*

**Table S10. Behaviours Targeted and Depth of BCTs Trained in Face-to-Face Training of Provider D**

| **Behaviour change technique** | **Depth of training** | **Diet** | **PA** | **Alcohol** | **Smoking** | **Unspecified** | **Other** |
| --- | --- | --- | --- | --- | --- | --- | --- |
| Action planning [1.4] | Directed | 0 | 1 | 0 | 0 | 1 | 0 |
| Action planning [1.4] | Instructed | 0 | 0 | 0 | 0 | 2 | 0 |
| Behavioural practice/rehearsal [8.1] | Instructed | 0 | 0 | 0 | 0 | 2 | 0 |
| Behavioural practice/rehearsal [8.1] | Demonstrated/ modelled | 0 | 1 | 0 | 0 | 0 | 0 |
| Behaviour substitution [8.2] | Instructed | 2 | 0 | 0 | 0 | 0 | 0 |
| Behaviour substitution [8.2] | Demonstrated | 1 | 0 | 0 | 0 | 0 | 0 |
| Biofeedback [2.6] | Demonstrated/ practiced/ modelled | 0 | 0 | 0 | 0 | 2 | 0 |
| Feedback on outcome(s) of behaviour [2.7] | Directed | 0 | 0 | 0 | 0 | 2 | 0 |
| Feedback on outcome(s) of behaviour [2.7] | Demonstrated/ practiced/ modelled | 0 | 0 | 0 | 0 | 2 | 0 |
| Goal setting (behaviour) [1.1] | Instructed | 0 | 1 | 0 | 0 | 0 | 0 |
| Goal setting (behaviour) [1.1] | Practiced | 1 | 1 | 0 | 0 | 0 | 0 |
| Goal setting (outcome) [1.3] | Practiced | 0 | 0 | 0 | 0 | 1 | 0 |
| Goal setting (outcome) [1.3] | Modelled | 0 | 0 | 0 | 0 | 1 | 0 |
| Habit formation [8.3] | Instructed | 0 | 0 | 0 | 0 | 2 | 0 |
| Increase positive emotions [x] *^a^* | Instructed | 0 | 0 | 0 | 0 | 2 | 0 |
| Information about emotional consequences [5.6] | Directed | 0 | 1 | 0 | 0 | 0 | 0 |
| Information about health consequences [5.1] | Directed | 0 | 2 | 0 | 0 | 0 | 0 |
| Information about health consequences [5.1] | Instructed | 4 | 1 | 0 | 0 | 0 | 2 |
| Information about health consequences [5.1] | Practiced | 1 | 0 | 0 | 0 | 0 | 0 |
| Problem solving [1.2] | Directed | 0 | 0 | 0 | 0 | 1 | 0 |
| Problem solving [1.2] | Instructed | 0 | 0 | 0 | 0 | 1 | 0 |
| Salience of behaviours [x] *^b^* | Demonstrated | 1 | 0 | 0 | 0 | 0 | 0 |
| Salience of behaviours [x] *^b^* | Modelled | 1 | 0 | 0 | 0 | 0 | 0 |
| Salience of consequences [5.2] | Instructed | 1 | 0 | 0 | 0 | 0 | 0 |
| Self-monitoring of behaviour [2.3] | Directed | 3 | 1 | 0 | 0 | 0 | 0 |
| Self-monitoring of outcome(s) of behaviours [2.4] | Instructed | 0 | 0 | 0 | 0 | 1 | 0 |
| Social support (unspecified) [3.1] | Directed | 0 | 0 | 0 | 0 | 1 | 0 |
| Social support (unspecified) [3.1] | Instructed | 0 | 2 | 0 | 0 | 6 | 0 |
| Social support (unspecified) [3.1] | Practiced | 0 | 0 | 0 | 0 | 1 | 0 |
| Social support (unspecified) [3.1] | Demonstrated/ practiced/ modelled | 0 | 0 | 0 | 0 | 1 | 0 |

*Note. Numbers in square brackets are corresponding number in BCTTv1. BCTs which were trained once but via multiple depths of training are shown across one row (e.g. ‘demonstrated/ practiced/ modelled’)*

*^a^ Increase positive emotions is not listed in the BCTTv1, but was noted by the authors for inclusion in the next version of the taxonomy.*

*^b^ Salience of behaviours was not listed in the BCTTv1, but has been identified as a new behaviour change technique by the authors of this paper.*
